# Crystal structure of Z-DNA in com­plex with the polyamine putrescine and potassium cations at ultra-high resolution^1^

**DOI:** 10.1107/S2052520621002663

**Published:** 2021-05-06

**Authors:** Pawel Drozdzal, Miroslaw Gilski, Mariusz Jaskolski

**Affiliations:** a Center for Biocrystallographic Research, Institute of Bioorganic Chemistry, Polish Academy of Sciences, Poznan, Poland; b Department of Crystallography, Faculty of Chemistry, A. Mickiewicz University, Poznan, Poland

**Keywords:** dual-conformation backbone, biogenic polyamine, biological potassium com­plex, subatomic resolution, Z-DNA

## Abstract

The X-ray crystal structure of the d(CGCGCG)_2_/putrescine(2+)/K^+^ com­plex has been determined at 0.60 Å resolution. Stereochemical restraints were used only for the putrescinium dication, and 23 bonds and 18 angles of the Z-DNA nucleotides with dual conformation. The N atoms of the putrescine(2+) dication form three direct hydrogen bonds with the N7_G atoms of three different Z-DNA molecules, plus three water-mediated hydrogen bonds with cytosine, guanine and phosphate acceptors. A unique potassium cation was also unambiguously identified in the structure, albeit at a ∼0.5 occupation site shared with a water molecule, providing the first example of such a com­plex with Z-DNA. The K^+^ cation has coordination number of eight and an irregular coordination sphere, formed by four water molecules and four O atoms from two phosphate groups of the Z-DNA, including ligands present at fractional occupancy. The structural disorder of the Z-DNA duplex is manifested by the presence of alternate conformations along the DNA backbone. Comparison of the position and interactions of putrescine(2+) in the present structure with other ultra-high-resolution structures of Z-DNA in com­plexes with Mn^2+^ and Zn^2+^ ions shows that the dicationic putrescinium moiety can effectively substitute these metal ions for stabilization of Z-type DNA duplexes. Furthermore, this comparison also suggests that the spermine(4+) tetracation has a higher affinity for Z-DNA than K^+^.

## Abstract 2

An ultra-high-resolution crystal structure of Z-DNA with the sequence d(CGCGCG)_2_ reveals the pattern of putrescinium(2+) and K^+^ binding. The com­plete polyamine dication is visible in the electron density and assumes a position that allows for hydrogen-bond interactions with three Z-DNA duplexes. The K^+^ cation occupies a mixed K^+^/H_2_O site and forms an irregular coordination sphere interacting with water molecules and the O atoms of two phosphate groups of two Z-DNA molecules, with some of the ligands present at fractional occupancy.

## 1. Introduction   

Biogenic polyamine cations are essential for cell growth and differentiation, and their biochemical significance in a wide spectrum of physiological functions has been repeatedly reviewed (Gugliucci, 2004 ▸; Moinard *et al.*, 2005 ▸; Larqué *et al.*, 2007 ▸; Igarashi & Kashiwagi, 2010 ▸; Jastrzab *et al.*, 2016 ▸). Common com­pounds in this group include the di­amines 1,3-di­amino­propane(2+) (Dap^2+^), putrescine(2+) (Put^2+^) and cadaverine(2+) (Cad^2+^), the tri­amines spermidine(3+) (Spd^3+^) and norspermidine(3+), and the tetramine spermine(4+) (Spm^4+^). Put^2+^ and Cad^2+^ are the two most common biogenic di­amines in bacteria. For example, at a concentration of 10–30 m*M*, putrescine is the predominant polyamine in *Escherichia coli* (Cohen, 1997 ▸). Polyamines(*n*+) are involved in maintaining the cell-envelope integrity of bacteria. They are also crucial to the virulence phenotype of many bacterial pathogens (Shah & Swiatlo, 2008 ▸; Di Martino *et al.*, 2013 ▸). Moreover, polyamines(*n*+) are important resources for viruses, which evolved mechanisms to maintain, enhance or manipulate polyamine(*n*+) metabolism to support viral infection (Firpo & Mounce, 2020 ▸). For instance, Mounce *et al.* (2017 ▸) showed the enhancement of viral polymerase activity by polyamines(*n*+). Polyamines(*n*+) are important factors stabilizing nucleic acids and stimulating their replication; only ∼7–10% of the total cell content exists as free polyamines(*n*+), the vast majority remaining in association with nucleic acids (Gugliucci, 2004 ▸). It was also shown that both polyamines(*n*+) and metal polycations (even at low concentration) can stimulate B to Z conversion of DNA and promote DNA condensation (Ohishi *et al.*, 2008 ▸; Vijayanathan *et al.*, 2001 ▸). Generally, both polyamines(*n*+) and metal cations can interact with Z-DNA molecules in one of two different modes. Polyamine(*n*+) cations are found to bind to the O atoms of different phosphate groups either directly or through water-mediated bridges. In the second mode, the cations are coordinated simultaneously by the guanine bases from different helices and also by the O atoms of dual-conformation phosphate groups (Tomita *et al.*, 1989 ▸; Gao *et al.*, 1993 ▸).

Among all Z-DNA (Wang *et al.*, 1979 ▸) com­plexes, the largest number of crystal structures in the Protein Data Bank (PDB; Berman *et al.*, 2000 ▸) and Nucleic Acid Database (NDB; Narayanan *et al.*, 2014 ▸) is available for com­plexes with magnesium and sperminium cations (Table 8 in Drozdzal, 2014 ▸). Z-DNA com­plexes with Dap^2+^ (PDB ID 2f8w), Spd^3+^ (2elg and 293d), *N*-(2-aminoethyl)-1,4-di­aminobutane (292d), thermospermine(4+) (336d), trientine(4+) (1dj6) and *N* ^1^-{2-[2-(2-aminoethyl­amino)ethyl­amino]ethyl}ethane-1,2-di­amine (2ie1) are also deposited in the PDB. Besides the Mg^2+^ cation, there are PDB structures of Z-DNA or Z-DNA-RNA (Gilski *et al.*, 2016 ▸) duplexes with many other metal(*n*+) cations, including Ba^2+^, Mn^2+^, Cu^2+^, Zn^2+^, Cr^3+^, Co^3+^ and Ru^3+^. There is currently no structure, however, of a Z-DNA/K^+^ com­plex.

While the structure of the Put^2+^ cation has been thoroughly studied (Woo *et al.*, 1979 ▸; Bratek-Wiewiorowska *et al.*, 1986 ▸; Jaskólski, 1987 ▸; Pospieszna-Markiewicz *et al.*, 2007 ▸), including the structures of phosphate salts (Jaskólski *et al.*, 1986 ▸; Bartoszak & Jaskólski, 1990 ▸) and a study at helium temperature (Jaskólski & Olovsson, 1989 ▸), it is rather poorly characterized as a component of nucleic acid structures. The Put^2+^ dication was specified as a nucleic acid ligand only in nine structures deposited in the PDB, all of them corresponding to *E. coli* ribosome.

In this article, we describe a very-high-resolution crystal structure of Z-DNA with the sequence d(CGCGCG)_2_, which for the first time provides insight into the interaction of Z-DNA with Put^2+^ and K^+^ cations. We also discuss the questions of competition and effectiveness of polyamine and metal cations in interactions with Z-DNA.

## 2. Materials and methods   

### 2.1. Oligonucleotide synthesis, purification and crystallization   

The methods of synthesis, deprotection and purification of the oligo­deoxy­nucleotide have been described previously (Xia *et al.*, 1998 ▸; Drozdzal *et al.*, 2013 ▸). A 1.5 m*M* water solution of the DNA oligonucleotide with the self-com­plementary seq­uence d(CGCGCG) was heated at 338 K for 10 min and then annealed slowly to room temperature overnight. Single crystals of the d(CGCGCG)_2_/Put^2+^/K^+^ complex were grown at 292 K by the hanging-drop vapour-diffusion method by mixing a 2 µl nucleic acid solution and a 2 µl precipitating solution consisting of 10%(*v*/*v*) (+/−)-2-methyl-2,4-pentane­diol (MPD), 40 m*M* sodium cacodylate pH 6.0, 80 m*M* KCl, 12 m*M* NaCl and 14 m*M* putrescinium dichloride. The drops were equilibrated against 0.5 ml 80%(*v*/*v*) MPD. Crystals appeared within one week and grew to dimensions of 0.3 mm × 0.1 mm × 0.1 mm.

### 2.2. X-ray data collection and processing   

X-ray diffraction data for the Z-DNA/Put^2+^/K^+^ com­plex were collected to a resolution of 0.60 Å on the EMBL beamline P13 (Cianci *et al.*, 2017 ▸) of the PETRA III storage ring at DESY, Hamburg. The crystal was vitrified in a stream of cold nitro­gen gas at 100 K. The mother liquor served as the cryoprotectant solution. The diffraction data were collected in two passes using two wavelengths and the following crystal-to-detector distances, oscillation ranges and numbers of images: λ 0.6880 and 0.7293 Å, 134 and 134 mm, 0.5 and 0.1° and 360 and 1800, respectively. The diffraction data were indexed, integrated and scaled using the *XDS* package (Kabsch, 2010 ▸). The X-ray data statistics are summarized in Table 1 ▸.

### 2.3. Structure solution and refinement   

The structure was solved by molecular replacement using *PHASER* (McCoy *et al.*, 2007 ▸). The DNA part of the PDB structure 4hig, corresponding to our earlier model of the d(CGCGCG)_2_/Spm^4+^/Mn^2+^ com­plex (Drozdzal *et al.*, 2013 ▸), served as a molecular probe. At the initial stages of the refinement, the model was refined using *REFMAC5* (Murshudov *et al.*, 2011 ▸) from the *CCP4* program suite (Winn *et al.*, 2011 ▸). The final anisotropic refinement was carried out with *SHELXL* (Sheldrick, 2015 ▸) using the full resolution of the diffraction data. The details of the *SHELXL* refinement were the same as described for our previous Z-DNA structures (Drozdzal *et al.*, 2013 ▸, 2015 ▸), except for the use of the DISP instruction, which allows the definition of the dispersion and absorption coefficients of a particular element without having to use the full format of the SFAC instruction. The instruction OMIT was used to exclude ten of the most disagreeable reflections (error/e.s.d. > 10) from the refinement. The model was validated using the *NuCheck* program (Feng *et al.*, 1998 ▸) and the free *R* test (Brünger, 1992 ▸), with 1876 reflections selected at random and set aside for *R*_free_ calculations.

It should be noted that the atomic scattering factor for the K^+^ site was declared on the SFAC instruction by specifying a neutral K atom. This small inaccuracy means assigning one excess electron per 18 actual electrons. This should have very little, if any, effect on the refinement, possibly under­estimating the refinable occupancy of the K^+^ cation by a factor of 18/19.

**Figure 1:**
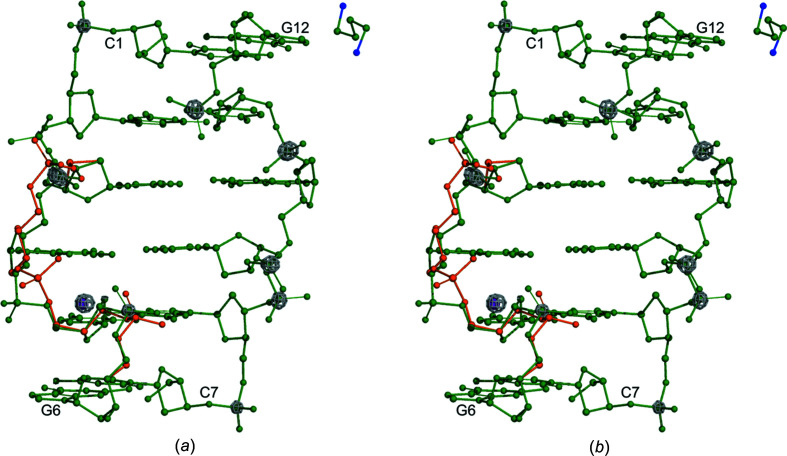
Stereoview of the d(CGCGCG)_2_/Put^2+^/K^+^ com­plex with anomalous difference map (gray) peaks for the K^+^ ion (purple sphere) and P atoms. The map is contoured at 3σ. Note the alternative conformations (I is green and II is orange) along the DNA chain.

At the wavelengths used in the diffraction experiments (0.6880, 0.7293 Å), the imaginary component of the anomalous scattering (*f*′′) of K and P atoms are, respectively, 0.236, 0.286 and 0.095, 0.104 electron units (Cromer, 1983 ▸). The anomalous signal is significant in the diffraction data set up to ∼0.9 Å resolution, as illustrated by clear peaks at the K^+^ ion and P atoms in the anomalous electron-density map (Fig. 1 ▸).

At this resolution, no stereochemical restraints are necessary to supplement the experimental observations (Jaskolski, 2017 ▸). However, restraints may still be needed for some disordered or highly mobile fragments. In the present structure, restraints were applied only to the putrescinium dication and to bonds and angles (33 for the sugar and eight for the phospho­diester moieties) of dual-conformation Z-DNA fragments. The ideal geometry targets for Put^2+^ were taken from Pospieszna-Markiewicz *et al.* (2007 ▸). Conformation-dependent geometrical restraints on bond lengths (DFIX) and bond angles (DANG) for the polynucleotide chains were generated using the *RestraintLib* server (http://achesym.ibch.poznan.pl/restraintlib/) as described by Kowiel *et al.* (2016 ▸, 2020 ▸) and Gilski *et al.* (2019 ▸). The CSD-derived conformation-dependent *RestraintLib* dictionary supersedes the classic nucleic acid restraints compiled by Parkinson *et al.* (1996 ▸). The final cycles of CGLS (conjugate-gradient least-squares) refinement converged with *R*/*R*_free_ values of 8.88/9.50%. The very last round of refinement, calculated with the test reflections included in the working set, converged with *R* = 8.77%. In order to provide estimations of standard uncertainties in all individual refined parameters and of all derived geometrical parameters, at the final stage of the refinement, one cycle of full-matrix least-squares minimization was calculated. Model placement in the unit cell was standardized with the *Achesym* server (Kowiel *et al.*, 2014 ▸).

The final model includes all the H atoms of the oligonucleotides and Put^2+^ added as riding contributions to *Fc*. The ammonium –NH_3_^+^ groups of Put^2+^ were refined using the instruction AFIX 33. The rotatable O3′/5′—H groups of the terminal sugars were refined using the instruction AFIX 87.

Figures presenting the model and electron density were prepared with *PyMOL* (DeLano, 2002 ▸). The *Coot* (Emsley *et al.*, 2010 ▸) program was used for visualization of the electron-density maps and manual rebuilding of the atomic model. The program *3DNA* (Lu & Olson, 2003 ▸) was used to calculate the Z-DNA helical parameters. The pseudorotation parameters were calculated by the method of Jaskólski (1984 ▸) using the *PseudoRotation* server (http://www.cryst.ump.edu.pl/pseudorotation.php).

### 2.4. Data deposition in public repositories   

Atomic coordinates and anisotropic ADPs, as well as the processed structure factors corresponding to the final model presented in this work, were deposited in the PDB with accession code 7atg. Raw X-ray diffraction images were deposited in the Integrated Resource for Reproducibility in Macromolecular Crystallography repository (proteindiffraction.org; Grabowski *et al.*, 2016 ▸) with DOI https://dx.doi.org/10.18430/m37atg.

## 3. Results and discussion   

### 3.1. Quality of the results   

The estimated standard uncertainties (e.s.u.) of the fully occupied DNA atomic positions in the structure are in the range 0.003–0.01 Å for C atoms, 0.003–0.007 Å for N atoms, 0.003–0.009 Å for O atoms and 0.001–0.002 Å for P atoms. The e.s.u. values for the full-occupancy covalent bonds are ∼0.005, ∼0.004, ∼0.004 and ∼0.003 Å for C—C, C—O, C—N and P—O, respectively. The agreement with stereochemical standards (r.m.s.d.) is 0.010 Å for bond lengths and 1.56° for bond angles. These results are comparable in terms of accuracy and precision with the record-setting model of Z-DNA (0.55 Å, *R* = 7.77%, PDB ID 3p4j) described by Brzezinski *et al.* (2011 ▸).

### 3.2. Overall structure and helical parameters   

The overall structural parameters of the DNA moieties of the d(CGCGCG)_2_/Put^2+^/K^+^ com­plex classify them within the Z-DNA family. The average helical twist Ω_h_ (*i.e.* the angle of rotation about the helical axis that brings successive base pairs into coincidence) per CG/GC tandem of base pairs is −60°. Other average base-pair and base-pair-step parameters are as follows: helical rise 4.37 Å, inclination (η) 14.55°, tip 2.6°, tilt 0.44°, roll −3.32°, shift −0.02 Å, slide 2.77 Å and rise 3.45 Å. Comparison of these helical parameters and base-pair geometries for the present and previously described Z-DNA com­plexes shows that they are within the range typical for Z-DNA duplexes (see supplementary Table S3 in Drozdzal *et al.*, 2015 ▸).

The 5′-d(CGCGCG) nucleotides of chain A in the asymmetric unit are numbered 1–6 and the com­plementary 3′-d(GCGCGC) nucleotides of chain B are numbered 12–7. There are only five internucleotide phospho­diester groups on each strand, which are numbered, respectively, P2–P6 and P12–P8. Alternative conformations are clearly visible in the electron-density map and are designated as I/II at the C3–G4, G4–C5 and C5–G6 internucleotide phosphate linkages, whose refined occupancies converged at 0.687 (8)/0.313 (8), 0.710 (4)/0.290 (4) and 0.650 (12)/0.350 (12), respectively. The major (I) and minor (II) conformations of C3 have C2′-*endo* and C1′-*exo* sugar puckers with pseudorotation (P) angles of 162.0 (7) and 138.2 (6)°, respectively. Differences in sugar pucker are also observed at G4, where conformation I is C3′-*endo* [P = 20.2 (7)°] and conformation II is C4′-*exo* [P = 38 (1)°]. Moreover, the *mFo–DFc* electron-density maps indicate three additional discrete positions (peaks) for the phosphate atoms at the C3 (6.7σ), C9 (6.0σ) and C11 (6.7σ) nucleotides. However, additional phosphate groups placed at these peaks refined with an occupancy below 0.20. Therefore, no atoms were modelled to interpret the peaks near the P_C3, P_C9 and P_C11 atoms. No alternative conformations were observed for the base moieties.

In the present com­plex, the sugars at the 3′-termini do not have the alternating C2′-*endo*/C3′-*endo* pucker of the pyrimidine/purine nucleotides, as is typical for Z-DNA, but all assume the C2′-*endo* conformation. The ZII conformation of the phosphate group can be assigned only to G4(I) with ζ = 64.6° [ζ is a backbone torsion angle defined as: C3′—O3′—P(*i* + 1)—O5′(*i* + 1)]. This ZII conformation is stabilized by a water-mediated OP2(I)_G4⋯Wat81⋯N2_G4 hydrogen bond.

### 3.3. Coordination of the polyamine cation   

**Figure 2:**
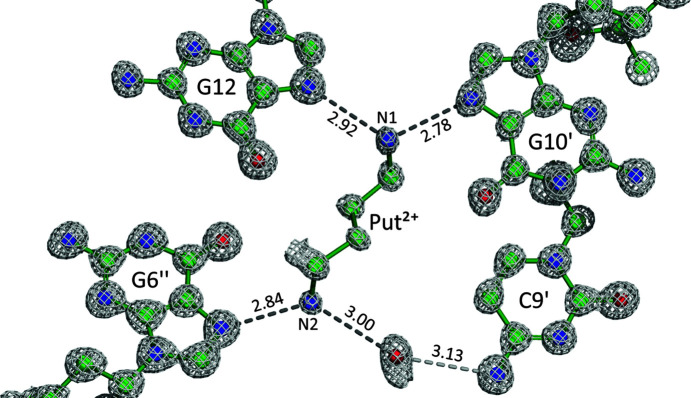
The putrescinium(2+) dication in the crystal structure of the d(CGCGCG)_2_/Put^2+^/K^+^ com­plex. The 2*mFo-DFc* map is contoured at the 1.6σ level. Some water molecules have been omitted for clarity. Hydrogen bonds are marked by dashed lines, with *D*⋯*A* distances in Å.

The entire putrescinium dication is clearly visible in the electron-density map (Fig. 2 ▸) despite its fractional occupancy, which converged on refinement at 0.378 (7). The putrescinium dication has a *gauche* ^−^–*trans*–*gauche* ^+^ conformation, with the following torsion angles: −72.5 (7), 172.9 (5) and 71.0 (8)°. The Put^2+^ dication is involved in direct hydrogen-bond contacts only with the guanine N7 atoms of three DNA duplexes (Fig. 2 ▸). The N1 atom is anchored by the N7 atoms of two neighbouring DNA duplexes at the following distances: N1_Put^2+^⋯N7_G12 = 2.915 (6) Å and N1_Put^2+^⋯N7_G10^i^ = 2.755 (6) Å [symmetry code: (i) *x* − 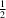, −*y* + 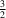, −*z* + 1]. The N2 atom is hydrogen bonded to N7_G6^ii^ at a distance of 2.839 (7) Å [symmetry code: (ii) −*x* + 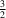, −*y* + 1, *z* − 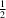]. The remaining three N—H donors of the putrescinium dication form water-mediated hydrogen bonds with the O6_G10^i^, N4_C9^i^ and OP1(I)_C5^ii^ atoms of two Z-DNA duplexes.

The electron-density maps indicate six water molecules in the vicinity of the Put^2+^ dication as alternative species populating the polyamine(2+) site at com­plementary occupancy. It should be noted that there was a ∼20-fold molar excess of Put^2+^ relative to the Z-DNA duplex in the crystallization mixture. The low occupancy of the Put^2+^ site is, therefore, not the result of insufficient supply of the ligand, but rather reflects the natural equilibrium of components required for growing those high-quality (from the point of view of diffraction quality) crystals.

There is very little literature information on putrescine(2+) interactions with longer d(CG)_*n*_ sequences. Putrescine had no effect on the conformation of a plasmid (pDHg16) with a 23-base pair d(GC)_23_ insert up to a 3 m*M* concentration (Thomas *et al.*, 1991 ▸). Our results indicate that the putrescine(2+) cation has preference for interactions with Z-DNA bases, which may explain why a 3 m*M* putrescine concentration was not sufficient for the B- to Z-DNA transition in the above plasmid. For longer d(GC)_*n*_ sequences *in vitro*, high concentrations of the putrescine(2+) cation may be needed to lower the energetic cost of Z-DNA formation.

### 3.4. Coordination of the K^+^ cation   

The electron-density maps clearly revealed one metal co­ordi­nation site with an occupancy of 0.49 (3), interpreted as potassium. Due to the partial occupation of the potassium cation, a com­plementary water molecule (Wat202) was also modeled in the 2*mFo–DFc* map at this site with an occupancy of 0.34 (6). After the refinement of this model, the *R* factor was reduced from 8.82 to 8.77%. The refined distance between the K^+^ and Wat202 sites is 0.195 (14) Å.

The metal was unambiguously identified as potassium using the following pieces of evidence. The length of the *M*—O bonds supports the presence of K^+^ rather than, for example, the presence of Na^+^ at higher occupancy. In the anomalous difference map, its peak (6.1σ) had a height similar to that of a full-occupancy P atom (P_11 at 6.4σ). The bond-valence method of Brese & O’Keeffe (1991 ▸), which correlates bond valences with the identity of the metal atom, is a popular method in coordination chemistry, and is especially reliable at high resolution. The application of this method gives values of the valence (*V*_K_) and bond-valence (*R*_KO_) parameters of 1.18, 1.09 and 2.07, 2.10 (the expected values for K^+^ being *V*_K_ = 1.00 and *R*_KO_ = 2.13) for the coordination sphere including Wat110(I)/Wat110(II), respectively (*vide infra*). Also, the application of the CBVS method of Müller *et al.* (2003 ▸) confirmed the identity of the metal site as K^+^ (calcium bond-valence sum CBVS = 0.53; according to the CBVS method, the reference value for K^+^ is 0.64). Finally, the *CheckMyMetal* server (Zheng *et al.*, 2014 ▸) also predicted potassium as the most likely cation at this site.

**Figure 3:**
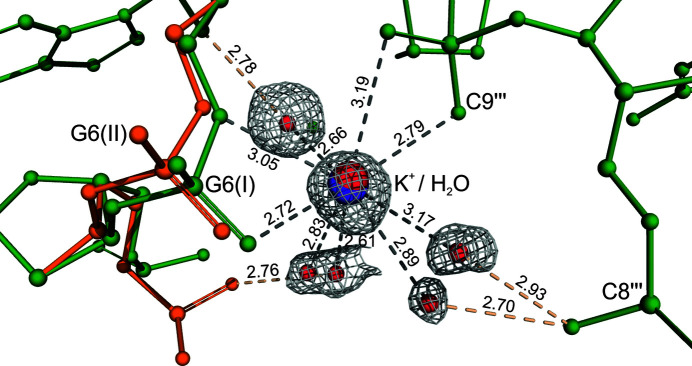
The coordination sphere of the hydrated com­plex of K^+^ (purple) at G6. The 2*mFo–DFc* map is contoured at 1.0σ. Coordination bonds are marked as gray dashed lines and hydrogen bonds are marked as orange dashed lines. Bond distances are in Å.

The K^+^ cation is located between two Z-DNA phosphate groups. There are eight O atoms (four from Z-DNA backbone and four from water molecules) in the immediate vicinity (up to a distance of 3.20 Å) of the K^+^ cation (Table 2 ▸). However, one of the water molecules (Wat110) and one phosphate group (OP1_G6) interacting with the potassium cation are disordered and were modelled in two com­plementary positions. In the presence of the OP1(I) and O5′(I) atoms (CN = 8), the coordination sphere can be considered as highly distorted square antiprismatic or dodecahedral. The K^+^ ion is coordinated simultaneously by OP1(I), O5′(I) from G6 and OP1, OP2 from C9^iii^ [symmetry code: (iii) −*x* + 1, *y* − 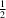, −*z* + 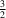], as well as by four water sites, one of which (Wat110) has dual occupancy (Fig. 3 ▸ and Table 2 ▸). The K^+^—O bond distances are in the range 2.612 (22)–3.185 (11) Å. The angles within the coordination sphere are irregular (Table 2 ▸).

### 3.5. Hydration   

The asymmetric unit contains 123 water sites. All water molecules were refined anisotropically without positional restraints. There was no attempt to model the H atoms of the water molecules. While for some water molecules it might be possible to try to locate their H atoms from difference Fourier maps, such H atoms have notoriously very poor geometry, even in small-molecule structures, and in macromolecular structures the dubious gain from their inclusion in the model usually does not compensate the burden of their individual handling in the refinement (Jaskolski, 2017 ▸). Summation of all the water occupancies in the asymmetric unit gives a total water content of 93.15. The positions of many disordered water molecules are correlated with the alternate I/II conformations of the Z-DNA backbones. With respect to their occupancy parameters, those water molecules for which the occupancies converged on refinement to values close to unity (>0.93) had their occupancy fixed at 1.0 (22 sites). Close pairs of sites for which the sum of their refined occupancies converged close to unity (36 pairs) had their combined occupancy constrained to 1.0. The remaining 65 sites had their occupancies refined freely to fractional values (all ≥0.20). The common patterns of solvent structure, as noted previously for Z-DNA crystals (Drozdzal *et al.*, 2013 ▸, 2015 ▸), such as water molecules between N2_G and phosphate O atoms, the spine of hydration (Chevrier *et al.*, 1986 ▸), two water molecules hydrogen bonded to each O6_G group or the absence of water molecules hydrogen bonded to the N3_G atoms, are also ob­served in the present Z-form structure.

## 4. Discussion   

In this work, we have presented a new crystal structure of Z-DNA/polyamine(*n*+), in com­plex with putrescine(2+) and K^+^ cations. It describes the first example of the interactions of putrescine(2+) with a DNA duplex. It is also the first case of Z-DNA crystallized in a com­plex with potassium ions. Moreover, the structure has the highest resolution and accuracy of the refined parameters among all DNA com­plexes with biogenic polyamines and/or metal cations deposited in the PDB.

**Figure 4:**
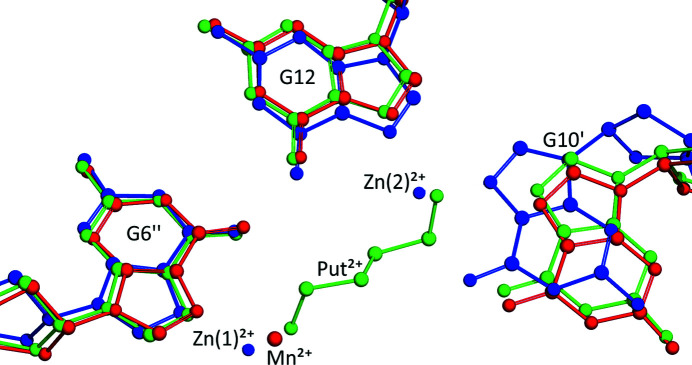
The d(CGCGCG)_2_/Spm^4+^/Mn^2+^ (red), d(CGCGCG)_2_/Spm^4+^/Zn^2+^ (blue) and d(CGCGCG)_2_/Put^2+^/K^+^(green) com­plexes in their common reference frame (unit cell). The interactions of the Mn^2+^, Zn^2+^(1) and Zn^2+^(2) ions and of the putrescinium(2+) N atoms with N7_G6, N7_G6^ii^ and N7_G10^i^ are indicated. A different location of G10^i^ in the com­plex with Zn^2+^ cations is also visible.

Although the crystallization systems for all the Z-DNA com­plexes presented in our previous studies (Drozdzal *et al.*, 2013 ▸, 2015 ▸) always contained KCl at a concentration of 40–80 m*M* in the crystallization drop, the K^+^ cation has been identified in the electron-density maps only in the present Z-DNA structure in com­plex with putrescine(2+). Comparison of the Z-DNA/Spm^4+^/Mn^2+^ and Z-DNA/Spm^4+^/Zn^2+^ structures with the present Z-DNA/Put^2+^/K^+^ com­plex in their common unit cell shows that the N1^+^ atoms of Spm^4+^ coincide almost exactly with the site of the K^+^ cation. This may indicate that the Spm^4+^ cation has a higher affinity for Z-DNA binding than the K^+^ cation and is preferentially selected when both are present in the crystallization buffer. It should also be stressed that the Z-DNA/K^+^ interaction has not been described in the literature so far in any crystallographic studies of Z-DNA structures. It is interesting to note that the general location of the N1^+^ and N2^+^ atoms of the putrescinium(2+) dication in the unit cell of the Z-DNA/Put^2+^/K^+^ com­plex is analogous to the positions of certain divalent metal cations, such as Mn^2+^ (PDB ID 4hig) or Zn^2+^(1) and Zn^2+^(2) (4hif) (Drozdzal *et al.*, 2013 ▸) (Fig. 4 ▸). Therefore, Put^2+^, having the same net charge as Mn^2+^ or Zn^2+^, can effectively replace these cations in interactions with the Z-DNA duplex. It is also worth mentioning that putrescine(2+) has a com­pletely different interaction pattern than 1,3-di­amino­propane(2+) (Dap^2+^) and 1,5-pentanedi­amine(2+) [cadaverine(2+)]. The structure of Z-DNA with Dap^2+^ (PDB ID 2f8w) indicates that this one-carbon-link-shorter polyamine interacts only with the phosphate groups of the Z-DNA (Narayana *et al.*, 2006 ▸). In a similar way, cadaverine(2+) (one carbon longer than Put^2+^) exhibits a preference for binding with Z-DNA phosphates only (Drozdzal *et al.*, to be published).

The putrescinium dication has been characterized in several crystal structures of its salts and in some of them it has the *gauche* ^−^–*trans*–*gauche* ^+^ conformation, while in the remaining cases it is all-*trans* (Woo *et al.*, 1979 ▸; Jaskólski *et al.*, 1986 ▸; Pospieszna-Markiewicz *et al.*, 2007 ▸).

Our study provides insight into the effectiveness and competition of polyamine and metal cations for interactions with Z-DNA, and confirms that even a simple di­amine can adopt different conformations and consequently enter into a variety of interactions with biomacromolecular partners.

The partial occupancy of the K^+^ ion confirms previous research findings suggesting that most observed monovalent cation sites in DNA crystals are partially occupied positions (Tereshko *et al.*, 2001 ▸; Dong, 2003 ▸). These cations are mobile and easily exchange sites with water molecules. This result also agrees both with (i) the conclusion from molecular-dynamic simulations which suggested that the structures of monovalent counter-ions in DNA are dynamic (Young *et al.*, 1997 ▸) and with (ii) the crystallographic studies on which the hybrid solvent model for the solvent structure around DNA is based and in which the solvent sites are occupied by water–cation hybrids (Shui *et al.*, 1998 ▸).

## Supplementary Material

PDB Validation Report. DOI: 10.1107/S2052520621002663/aw5052sup1.pdf

Raw X-ray diffraction data: https://dx.doi.org/10.18430/m37atg

## Figures and Tables

**Table 1:** Data collection and refinement statistics for d(CGCGCG)_2_/Put^2+^/K^+^

Data collection	
Radiation source	P13, Petra III, DESY, Hamburg
Wavelength (Å)	0.6880, 0.7293
Temperature (K)	100
Space group	*P*2_1_2_1_2_1_
Cell dimensions (Å)	*a* = 17.97, *b* = 31.02, *c* = 43.86
Resolution range (Å)	25.33–0.60 (0.61–0.60)^*a*^
Number of reflections	107 989^*b*^
Completeness native (%)	90.5 (22.6)
Redundancy	3.7 (1.2)
〈*I*/σ*I*〉	29.8 (2.9)
CC_1 \over 2_^*c*^	99.9 (81.1)
*R*_merge_^*d*^ (%)	2.4 (26.4)
Wilson *B*-factor (Å^2^)	5.17
	
Refinement	
Refinement program	*SHELXL* (Sheldrick, 2015 ▸)
Resolution (Å)	25.33–0.60
No. of reflections in working set	106 103^*b*^
No. of reflections in test set	1876
*R*, *R*_free_^*e*^ (%)	8.77, 9.50
No. of atoms (nucleic acid, solvent, polyamine, metal)	240, 123, 6, 1
〈*B*〉 (Å^2^) (nucleic acid chain A, B, solvent, polyamine, metal)	4.56, 3.99, 11.93, 4.27, 6.56
R.m.s. deviations from ideal bond lengths (Å), angles (°)	0.010, 1.56

Notes: (*a*) values in parentheses correspond to the last resolution shell; (*b*) Bijvoet pairs separated; (*c*) correlation between intensities from random half-sets as defined by Karplus & Diederichs (2012 ▸); (*d*) *R*_merge_ = Σ_*h*_Σ_*j*_ |*I*_*j*_ − 〈*I*〉|/Σ_*h*_Σ_*j*_ *I*_*j*_, where *I*_*j*_ is the intensity of observation *j* of reflection *h*; (*e*) *R* = Σ_*h*_||*F*_o_| − |*F*_c_||/Σ_*h*_|*F*_o_| for all reflections, where *F*_o_ and *F*_c_ are the observed and calculated structure factors, respectively. *R*_free_ was calculated analogously for the test reflections, which were randomly selected and excluded from the refinement.

**Table 2:** Coordination geometry (Å, °) around the metal ion in the d(CGCGCG)_2_/Put^2+^/K^+^ structure, with standard uncertainties in parentheses

	Distance	Angles							
W109	2.661 (8)								
W110(I)	2.612 (22)	80.3 (6)							
W110(II)	2.831 (39)	72.3 (10)	12.4 (8)						
W111	2.887 (20)	157.3 (8)	77.8 (8)	87 (1)					
OP1(I)_6	2.715 (8)	108.8 (2)	97.1 (7)	90.1 (10)	80.0 (4)				
O5′(I)_6	3.047 (11)	73.4 (2)	123.6 (6)	111 (1)	124.5 (6)	49.5 (2)			
W117	3.172 (29)	168.0 (5)	107.4 (7)	117 (1)	29.8 (6)	79.9 (3)	108.3 (5)		
OP1_9^iii^	3.185 (11)	80.4 (2)	151.4 (7)	150.7 (9)	117.3 (5)	109.1 (4)	69.9 (3)	89.0 (5)	
OP2_9^iii^	2.792 (7)	88.3 (2)	108.6 (7)	118 (1)	93.1 (4)	151.4 (5)	119.2 (4)	80.5 (4)	49.9 (2)
-	K^+^	W109	W110(I)	W110(II)	W111	OP1(I)_6	O5′(I)_6	W117	OP1_9^iii^

Symmetry code: (iii) −*x* + 1, *y* − 1 \over 2, −*z* + 3 \over 2.
